# How to capitalize on investors by using information presentation and feedback on crowdfunding projects

**DOI:** 10.3389/fpsyg.2022.831333

**Published:** 2022-08-05

**Authors:** Zhaoxiang Wu, Shaojun Yan, Jilin Dai

**Affiliations:** ^1^Business School, Sun Yat-sen University, Guangzhou, China; ^2^School of Business Administration, Tongling University, Tongling, China

**Keywords:** information presentation, information feedback, signal theory, crowdfunding experience, financing performance

## Abstract

As an innovative financing activity, online crowdfunding is characterized by extremely high information asymmetry. To reduce this information asymmetry, crowdfunding companies typically use information presentation, feedback, and other means to convey more information about the fundraising project to investors. Whether the information presentation and feedback affect the investment behavior of nonprofessional ordinary investors is yet to be determined. Moreover, the method by which the information presentation and feedback influence the investment behavior and consequently, the financing performance of crowdfunding companies, has to be identified as well. Currently, research on this subject remains deficient. Therefore, with signal theory and the difference in the cost of information transmission considered, this study classifies the information released by fundraisers on the crowdfunding platform into two categories: low-quality signal and high-quality signal. Projects on the JD.com Crowdfunding website are then used as research samples to explore how the difference in signal quality in the information presentation and feedback of crowdfunding projects influences financing performance from the perspective of investors. The results show that low-quality signals such as video duration, the number of updates, and the number of comments on projects positively affect the success of crowdfunding; meanwhile, crowdfunding experience, which represents high-quality signals, positively moderates the relationship between project video duration, project updates, and crowdfunding success.

## Introduction

Solving the problem of information asymmetry between fundraisers and investors is key to successful fundraising for startups (Mollick, 2014; Ribeiro-Navarrete et al., 2021). Owing to the existence of “Liability of Newness” and “Liability of Smallness,” new ventures are often excluded from traditional external financing channels (bank loans, venture capital, etc.). However, the development of the new financing model, particularly the “crowdfunding” model, which is based on the Internet platform and directly seeks financing from the public, provides new financing channels and opportunities for new start-ups (Sewaid et al., 2021). The COVID-19 outbreak in 2020 forced enterprises and individuals to undergo rapid digital transformation and constantly learn and improve their digital skills (Soto-Acosta, 2020). Digital transformation not only changes the decision-making tendency of investors but also provides an opportunity for startups to raise project funds through the digital platform. Kraus et al. (2019) predicted that as part of the digital economy, crowdfunding platforms will achieve rapid growth in the next several years. According to the China Crowdfunding Industry Development Report 2021, the annual amount of crowdfunding in China reached RMB 72.3 billion in 2021 and is soon expected to exceed venture capital and become the main provider of venture financing. This novel financing model has been recognized by the industry and has also attracted widespread attention from academies.

Despite the rapid development of crowdfunding financing, its inherent defects are also extremely apparent. (1) Serious asymmetry of information. In the crowdfunding financing model, the transaction is characterized by concealment and anonymity owing to the inability of the crowdfunding parties to conduct face-to-face communication, which impedes the ability of the investor and financing parties to obtain true information from each other (Tafesse, 2021); (2) Amateurism of investors. Crowdfunding investment participants are mainly ordinary investors, who have no professional investment knowledge and rich investment experience, in contrast to professional venture investors; moreover, they invest mostly based on their feelings or subjective preferences and thus are highly speculative (Herrero et al., 2019). The existence of these endogenous defects directly affects the financing performance of crowdfunding companies. With the shortcomings mentioned above, what actions the fundraisers should take to influence the investment decision-making and investment behavior of “amateur” investors and thus improve their financing performance?

Previous studies on signaling theory (Spence, 1978, 2002) conclude that providing high-quality signals is the primary approach to enhancing the financing performance of crowdfunding companies. With the uncertainty of crowdfunding investments, investors normally judge projects on the basis of the project information. High-quality signal information certainly boosts their investment confidence (Bapna, 2019; Kleinert et al., 2021). These high-quality signals include quality characteristics, including the financial and social status of the crowdfunding project (Allison et al., 2017); a description of the founder experience, the management system, and the equity allocation scheme of the startup; among others (Robb and Robinson, 2014). High-quality signals increase the investment preferences of investors, but this does not suggest that low-quality signal information is ineffective, particularly for amateur investors in crowdfunding. Li et al. (2017) find that investors make investment decisions based on their subjective impressions of the motivations and capabilities of entrepreneurs. This finding suggests that low-quality signals such as entrepreneurial enthusiasm and motivation can potentially exert a positive effect on investment decisions. Compared with traditional venture investors, amateur investors who participate in crowdfunding have difficulty obtaining objective information about companies and possess relatively insufficient investment experience (Liu et al., 2021). Therefore, these interest-driven amateur investors may rely more on low-quality signals from crowdfunding companies to make decisions. However, the existing research on crowdfunding financing has not paid considerable attention to the aforementioned question.

Drover et al. (2018) and Stern et al. (2014) indicate that signals rarely exist in isolation; instead, they usually interact with each other and act on objects together. Plummer et al. (2016) report that high-quality signals may weaken or counteract the effects of low-quality signals, and the synergy between high-quality and low-quality signals may reduce information asymmetry. Moreover, reducing information asymmetry and improving financing performance are key issues in crowdfunding financing. Thus, faced with numerous amateur investors, do crowdfunding companies (1) provide only high-quality signals to boost investor confidence; (2) provide only low-quality signals to lure investors with “spoken words”; or (3) not only provide high-quality signals to enhance investor confidence but also induce investors through “spoken words”? That is, under information asymmetry, among uneven fundraisers, do amateur investors prefer (1) crowdfunding companies that only provide high-quality signals; (2) crowdfunding companies that only provide low-quality signals to entice investors with “spoken words”; or (3) crowdfunding companies that do not only provide high-quality signals to enhance investor confidence but also induce investors through “spoken words”? The existing research provides no clear answer.

To compensate for the deficiency of the aforementioned research, this study categorizes the information released by fundraisers on crowdfunding platforms into high-quality signals and low-quality signals on the basis of the signal theory, as well as constructs a structural model between high-quality signals, low-quality signals, and crowdfunding performance of fundraisers to explore the mechanism underlying the influence of low-quality signals on financing performance and the moderating effect of high-quality signals. This study presents the following major contributions: First, the asymmetry of strategic decision-making information and entrepreneurial opportunity attributes are introduced into the crowdfunding field, which enriches the research on the influencing factors of crowdfunding performance. As a bilateral market, the crowdfunding platform has various types of projects, which are heterogeneous. In accordance with the creation and discovery theories of entrepreneurship, this study analyzes the response of the capital market to the entrepreneurial opportunities of exogenous and endogenous crowdfunding projects from the perspective of opportunity attributes. Second, as a financing method to support creative ideas, crowdfunding has the characteristics of “tasting fresh” and “emphasizing experience.” The information and signals contained in crowdfunding projects would inevitably affect crowdfunding performance. Under the circumstance of crowdfunding on the Internet, this study divides the information contained in crowdfunding into high-quality signals and low-quality signals from the perspective of investors, as well as evaluates its effect on financing performance, thus enriching the research on signal theory.

## Literature review

### Behavior and performance of crowdfunding

Alvarez and Barney (2007) indicated that the strategic decision-making behavior (including financing) comprehensively reflects market opportunities, entrepreneur characteristics, and the decision-making situation. Discovery theory holds that opportunities are exogenous and can be observed—that is, the risk may be predicted. However, owing to the information asymmetry and variations in risk preference or cognition among entrepreneurs (Shane and Delmar, 2004), these exogenous opportunities cannot be realized and utilized by everyone, resulting in differences in discovering and grasping opportunities for entrepreneurs. By contrast, creation theory holds that opportunities are endogenous and can be created by the actions of entrepreneurs to explore new products or services as well as market reactions (Baker and Nelson, 2005). However, such endogenous opportunities are difficult to recognize before they are created; thus, the risks are unpredictable.

The different risk characteristics of exogenous and endogenous opportunities lead to different reactions of the capital market. For the former, the capital market tends to agree on investment depending on the results of risk assessment; for the latter, the capital market often chooses to avoid it because its risk is difficult assess (Alvarez and Barney, 2007). Therefore, faced with endogenous opportunities, fundraisers usually rely on themselves, their friends, and their family for support to carry out activities. The reason these consanguineous (relative/school mates/geographic) investors invest in fundraisers is not excess optimism about the “opportunity”; rather, it is the trust or the confidence of investing in “this person” instead of investing in the “opportunity.” Therefore, investors generally "invest according to events” (rational investment originated from the assessable exogenous opportunity risk) or “invest according to person” (the perceptual investment originated from the network of acquaintance to reduce endogenous opportunity risk); alternatively, they invest according to both factors (exogenous opportunities originated from acquaintances, i.e., rationality plus perceptions) and basically does not invest for the fourth reason, that is, the endogenic opportunity that does not originate from an acquaintance. However, in the crowdfunding market, these four situations exist simultaneously.

Crowdfunding is a new financing channels in which fundraisers display creative or entrepreneurial information through an Internet platform and attract many investors to make small investments to support entrepreneurship or other activities. Crowdfunding performance refers to the overall achievements of crowdfunding projects from the start of financing to the completion of the financing process. It is mainly reflected in the number of financing, whether the financing objectives are achieved, and the completion percentage of financing objectives (Allison et al., 2017). The existing research focuses on various factors that affect crowdfunding performance (Short et al., 2017). One view is that the relationship network is an important factor influencing crowdfunding performance. Zheng et al. (2018)indicated that the social network of project sponsors exerted an important effect on financing performance; Colombo et al. (2015) considered the interaction among investors and between investors and initiators as key factors influencing crowdfunding performance. Another view is that the linguistic expression of the project or the purpose of the project can affect crowdfunding performance. According to Parhankangas and Renko (2017), project-based explanatory information indicates that language plays an important role in crowdfunding. Allison et al. (2017) showed that when the project sponsor emphasizes the charitable characteristics of the project, the investor has a positive willingness to invest; when the project sponsor emphasizes that it is a business opportunity, the willingness of the investor to invest is markedly low. Other key factors influencing crowdfunding performance include entrepreneurial ability and motivation, such as entrepreneurial passion (Li et al., 2017) and the positive psychological capital of fundraisers (Anglin et al., 2018).

Social network, language expression, or the charitable nature of the project, as well as entrepreneurial passion or psychological capital, are signals conveyed from fundraisers to investors. For crowdfunding enterprises, the means by which signals are conveyed and the signals to convey are key to financing success; for investors, identifying signals is vital.

### Signal attributes in crowdfunding

First proposed by (Spence, 1978), signal theory is mainly used to solve the core problem encountered by strategic decision-makers, that is, the use of various signals to reduce the uncertainty of choice caused by asymmetric and incomplete information (Spence, 2002). Signal theory has been widely applied in the existing research of entrepreneurship. According to Pabst and Mohnen (2021), signal theory is currently used in research to analyze how startups attract investment by transmitting organization quality signals to internal key stakeholders (such as investors).

Different signals vary in quality because of the different costs of acquiring and transmitting signals (Connelly et al., 2011). High-quality signals include project quality characteristics, financial status, social network founder experience, management system, and equity allocation scheme of the startup enterprise (Robb and Robinson, 2014; Allison et al., 2017), whereas low-quality signals include entrepreneurial enthusiasm and entrepreneurial motivation (Li et al., 2017; Anglin et al., 2018). Startup enterprises usually fail to provide high-quality signals because of the “Liability of Newness” and “Liability of Smallness” but have a high demand for financing. Therefore, the key to success for startup enterprises is the use of low-quality signal to solve the contradiction between congenital weakness and financing demand.

Danilov and Sliwka (2017) and Loewenstein et al. (2014) showed that when the company lacks objective information and a clear code of conduct (or high-quality signals) and investors lack investment expertise or experience, investors can use low-quality signals to evaluate the quality of a startup enterprise. Guillamon-Saorin et al. (2017) explicitly indicated that the language or statement that organization leaders use is a key signal to evaluate the quality of a company although the signal is of low quality. Despite the low cost these low-quality signals for fundraisers, other related costs may be incurred, such as reputation damage, legal costs, or customer churn (Payne et al., 2013).

As a type of entrepreneurship, crowdfunding enterprises also suffer from the “Liability of Newness” and the “Liability of Smallness.” They encounter difficulties providing high-quality signals to gain the favor of professional investors. Therefore, they appeal to the public investors or amateur investors who lack professional investment knowledge or experience and have a small investment scale but want to gain profits (Malmendier and Shanthikumar, 2007). Their small investment scale while crowdfunding enterprises that only meet their investment needs impede amateur investors from competing with professional investors in high-quality projects. Therefore, how to convey signal, what signal to be convey and how to screen signal is the critical solution for the contradiction between financing demands of crowdfunding companies and investment decisions of amateur investors, and also the important factors influencing performance of both crowdfunding companies and amateur investors.

## Theory and hypotheses

### Information feedback and crowdfunding performance

Reward-based crowdfunding is a major form of crowdfunding (Mollick, 2014; Ahlers et al., 2015). It mainly utilizes a “pre-sale” business model to attract mass investors of future product consumers. The product is not completely designed or produced before the success of crowdfunding. It merely exists in imagination, creativity, or design. Investors are unable to directly access the product, the quality of which is difficult to estimate. Fundraisers only use a structured narrative language to indicate their status or provide product information. Language information usually consists of three parts, including “project details, project updates, and comments.” The cost of providing this textual information is extremely low for fundraisers (Anglin et al., 2018), and the cost of transmitting information through the Internet is low (Bolton et al., 2004); thus, it is a low-quality signal. However, this kind of low-quality signal may be an important factor for amateur investors to consider in their investment decisions and may be a key element influencing crowdfunding performance.

On reward-based crowdfunding platforms (such as JD Crowdfunding), fundraisers present their projects to investors through the video content in “project details.” The presentation is intended to help investors thoroughly understand the market competition status, reputation, capabilities, and product quality, of fundraising companies in addition to the vision and commitment of fundraisers. The information is only a prerequisite for investor support and not a sufficient condition. To capitalize from amateur investors who are fragmented, relatively independent of one another, uncoordinated, and lacking in professional investment experience, the key is to ensure whether the low-quality signal provided by the fundraiser in the “project details” can truly impress investors or arouse their empathy.

The investment decision of an investor is usually influenced by the number of investment signals: the greater the number of investment signals, the stronger the impulse to invest (Anglin et al., 2018). This requirement can be fulfilled by the video content in “project details” on crowdfunding platforms. The video content in the “project details” is displayed and saved on the platform as a complete “project impression” signal to entice potential investors to pay attention to the projects and offer support at any time. Moreover, the video content in the “project details” serves as a construction tool for conveying meaningful signals, which can help potential investors elucidate the ideas, behaviors, and intentions of the entrepreneurs. Lastly, the video content in “project details” also demonstrates entrepreneurial qualities, such as entrepreneurial passion, to a certain extent, thus triggering emotional reactions from investors. Therefore, this article suggests that detailed project information containing all details of entrepreneurial activities is more likely to gain the approval and support of investors than simply persuading stakeholders to participate.

*Hypothesis 1*: On crowdfunding platforms, the more video content is made available in “project details,” the better the financing performance of crowdfunding companies.

Crowdfunding platforms continuously release project updates, apart from releasing video information of project details, during the crowdfunding period. This update summarizes the progress of the crowdfunding project from the fundraisers. The updated information of the project is an important guiding signal that helps fundraisers convey to investors the skills, information, and planning necessary to achieve subsequent financing goals, which is considerably useful for crowdfunding investors who do not rely on experience, habits, or expertise (Block et al., 2018). The financing goals of the fundraiser are self-determined based on the development of the new venture. Without the guidance of the past performance records of the company, the information used by investors for assessment comprises unattested signals (Wehnert et al., 2019). At this moment, the update signal of the project becomes more important as an external evaluation tool. Specifically, the higher the proportion of current financing in the financing target, the greater the value of the financing project (Courtney et al., 2017). Therefore, the more updated the project information, the smoother the progress of crowdfunding project financing. In this manner, the risk of project failure perceived by investors is reduced, and the trust of investors in the ability of fundraisers is increased, affecting the investment decisions of investors.

*Hypothesis 2*: On crowdfunding platforms, the faster the project progress is updated, the better the performance of corporate financing.

The comment section of the crowdfunding website not only allows fundraisers to release relevant information and communicate with investors but also enables communication between investors. Thus, the comment section of the project offers a bidirectional flow of information between fundraisers and investors. It is also a channel through which the strategy and product information of the new venture is communicated by fundraisers to investors, which helps investors obtain accurate information. With this information, investors can more directly understand the logic and potential risks associated with the products, enhancing their ability to explain the products to others (Jimenez and Mendoza, 2013). Moreover, two-way communication is not only a unilateral output of information but also a means for investors to give feedback to fundraisers, who can then promptly apply improvements and corrections. This communication prompts investors to become more inclined to support individuals or groups who are willing to follow advice and take the necessary steps to achieve their goals, rather than support fundraisers who seem to disregard suggestions from investors and lack commitment (Bender et al., 2019). In addition, the exchange of information in the comment section of the crowdfunding platform is beneficial to corporate financing regardless of whether the comment carries positive or negative content. The reason is that for crowdfunding projects, the corresponding products or services do not exist until the end of the crowdfunding project, and investors are not able to personally “experience” the actual quality of the project (Benedicktus et al., 2010). Consequently, the comments posted by investors in the comment section are merely intended to request the project sponsor for information about the project or to express their subjective attitude or views on the project (Shneor and Munim, 2019). The responses of fundraisers do not only address questions from the investors but also signal investors to evaluate the attitude of the sponsors, as well as their enthusiasm and diligence, which is also one of the essential bases on assessing project quality for potential investors and even finalize investment decisions accordingly.

*Hypothesis 3*: On crowdfunding platforms, the greater the number of project reviews, the better the performance of corporate financing.

### Moderating effect of crowdfunding experience

The previous assumptions mainly involve low-quality signals, such as the content of project information, the speed at which information is updated, and the information in the comment section. Plummer et al. (2016) indicated that on crowdfunding platforms investors have difficulty understanding the intrinsic meaning of signals sent by fundraisers because of information asymmetry. The reason is that the content on the crowdfunding platform is essentially information that is difficult to verify (Belleflamme et al., 2015), and any piece of information tends to be ambiguous because of the experience of the investors(GIOIA and CHITTIPEDDI Gioia and Chittipeddi, 1991). Although crowdfunding companies convey psychological signals such as confidence and tenacity to investors via project details, updates, and review information on the crowdfunding website, investors still tend to question the authenticity and accuracy of the information and prefer that fundraisers provide them as much information as possible to facilitate their investment decisions. Courtney et al. (2017) and Stern et al. (2014) suggest that fundraisers can elucidate low-quality signals by providing investors additional signal information comprising high-quality signals to boost investor trust and to further increase investment possibilities for investors.

Crowdfunding experience (that is, the experience accumulated in the previous crowdfunding entrepreneurship) is the “tacit knowledge” that fundraisers can use. Politis (2005) find that this crowdfunding experience can assist startups as they overcome new disadvantages. Fundraisers with crowdfunding experience often perform better than novices in crowdfunding activities and can better ensure the success of entrepreneurial activities (Colombo et al., 2015; Lafontaine and Shaw, 2016). The reason is that fundraisers have been access to different sectors of knowledge and make relations with different supporters through multiple crowdfunding activities to accumulate rich human capital and crowdfunding experience (Ko and McKelvie, 2018; Kleinert et al., 2021). Human capital includes rich industry experience and keen observation of entrepreneurial opportunities (Martin et al., 2013), and crowdfunding experience includes goodwill value earned from individual or collective social relations (Gedajlovic et al., 2013). Rich human capital and crowdfunding experience are gained by fundraisers through spending time, energy, and resources in repeated fundraising activities and thus has a high cost of investment. Moreover, abundant human capital and social capital can produce a spillover effect, indicating that others have made guarantees for fundraisers (Ahlers et al., 2015). Therefore, social capital and human capital are the key signals for investors in assessing the quality of financing projects (Grichnik et al., 2014). Similarly, the crowdfunding experience of the fundraisers is a high-quality signal.

Signal theory states that a high-quality signal is an important basis for investment decisions. The higher the quality of signals, the better the quality of the enterprise, the more valuable the future benefits, and the more predictable the returns and investment confidence for investors. Anglin et al. (2018) observed that when an enterprise transmits a series of signals simultaneously, an interaction occurs between signals, which can change the investor cognition of the enterprise. Generally, the project information content, update speed, and comment area information provided by crowdfunding enterprises on the crowdfunding platform are basically low-quality signals. Investors may express positive, negative or neutral views on these low-quality or low-cost signals because of differences in their comprehension ability, resulting in low investment expectations and wait-and-see behavior. Plummer et al. (2016) explained that confronted with the aforementioned situation, fundraisers can introduce high-cost signals to reduce the fuzziness and noise of low-quality signals, guide investors to better understand the enterprises to invest in, stimulate the endorsement effect (Honig et al., 2006), enhance the relationship between investors and the enterprises, and promote their investment interest. As a high-quality signal, the crowdfunding experience of the fundraiser can improve the interest and confidence of investors in crowdfunding projects (Bruns et al., 2008). The underlying logic is that the crowdfunding experience, particularly the continuous and successful crowdfunding projects, of the fundraiser proves not only that the fundraiser exhibits efficiency in operating the project but also that the fundraiser possesses rich human and social capital. These characteristics are highly valued by investors. In addition, the experiential verification by previous investors of the products, services, and goodwill of the crowdfunding projects attributed to the funders can undoubtedly serve as third-party endorsements of the funders, which can virtually enhance investor confidence (Bento et al., 2019). Therefore, the high-quality signal of crowdfunding experience can support other low-quality signals, enhance the authenticity and reliability of low-quality signals, elucidate the information expressed by crowdfunding enterprises, enhance the persuasion of enterprise value, and contributes to the improvement of enterprise financing performance.

*Hypothesis4*: The crowdfunding experience of the fundraiser has a moderating effect on the relationship between the descriptive information of the crowdfunding project and crowdfunding performance.

*Hypothesis 4a*: On crowdfunding platforms, the crowdfunding experience of the fundraiser has a moderating effect on the relationship between the information contained in the “project details” and the financing performance of the crowdfunding company.

*Hypothesis 4b*: On crowdfunding platforms, the crowdfunding experience of the fundraiser has a moderating effect on the relationship between the project progress update and the financing performance of the crowdfunding company.

*Hypothesis 4c*: On crowdfunding platforms, the crowdfunding experience of the fundraiser has a moderating effect on the relationship between the number of project reviews and the financing performance of the crowdfunding company.

**Figure 1:**
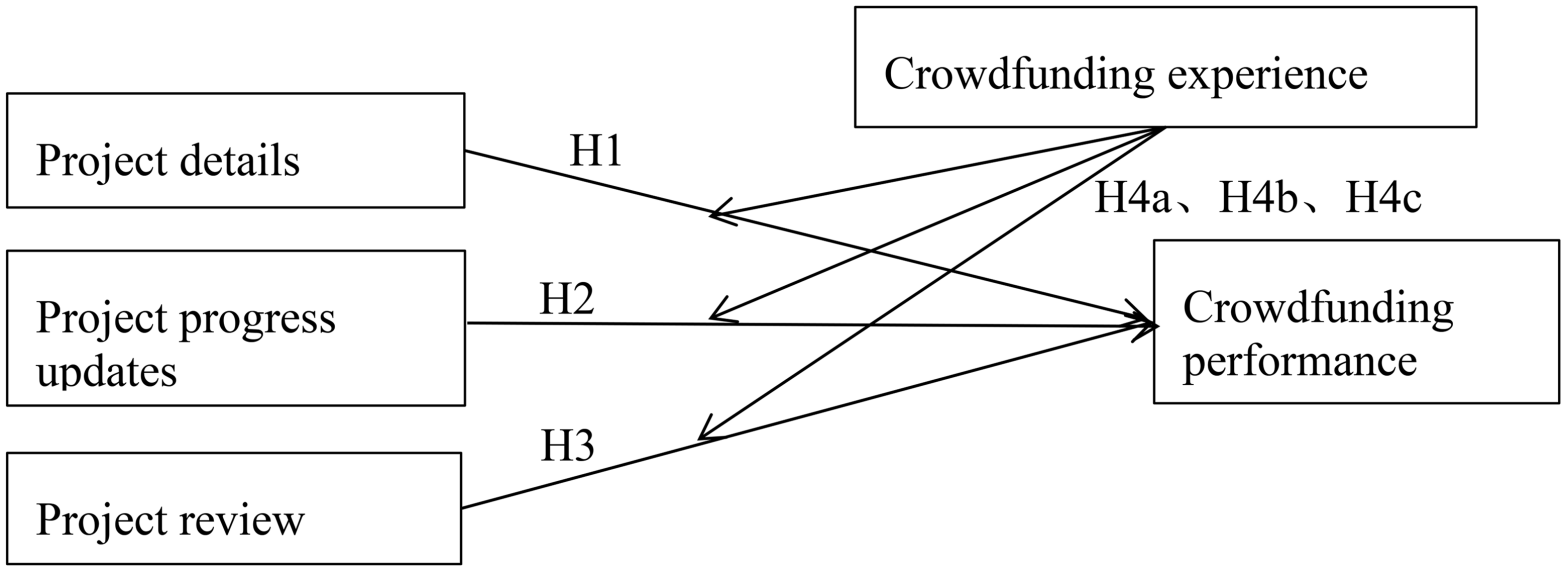
Conceptual model of the study.

Our conceptual model is summarized as Figure 1.

### Research design

#### Data source

In accordance with the availability and timeliness of the data, the research data used in this study come from the widely known crowdfunding website JD.com Crowdfunding.^1^ The reward-based crowdfunding on JD.com Crowdfunding mainly provides information such as project type, fundraiser, project details, progress, topic, project target amount, the amount raised, financing completion ratio, and comments, among others. After collecting data, this study received a total of 2,499 sample data from various fields, including technology, catering, home appliances, design, entertainment, culture, and public welfare, among others. After screening, samples with an excessive target amount were eliminated; ultimately, 2,283 sample data remained. During data collection, the relevant variable information from the JD.com Crowdfunding website was extracted manually.

#### Variable measurement

Similar to most studies, the current research considers the ratio of the actual fundraising amount to the target amount as the dependent variable (Tafesse, 2021). In other studies, the dependent variables are the number of supporters of the crowdfunding project (Bi et al., 2017) and the fundraising amount and the number of funders (Colombo et al., 2015). The independent variables refer to the video introducing the project, the number of updates to projects, and the number of comments on projects (Schlosser, 2011; Mollick, 2014). Specifically for the current study, the moderating variable is the crowdfunding experience of the fundraisers. Following previous crowdfunding studies, we use the number of crowdfunding projects initiated by entrepreneurs to measure this variable. By continuously launching projects on the crowdfunding platform, companies can obtain social capital within their crowdfunding communities (Buttice et al., 2017). The control variables selected mainly include the target fundraising amount, the number of likes, and the number of followers that have been proven to significantly affect financing performance in existing studies (Benedicktus et al., 2010).

#### Descriptive statistical analysis

This study includes 2499 crowdfunding projects from the JD Crowdfunding website. After excluding projects without specific termination time, 2283 crowdfunding projects within the standard crowdfunding period were retained. Table 1 lists the descriptive statistics for the crowdfunding projects. These data include, but are not limited to, the following: maximum fundraising amount (Goal) of crowdfunding projects, RMB 5 million; maximum number of likes (Focus), 80,000 individuals; and maximum number of followers of crowdfunding projects, 3000 persons. Moreover, 5 types of crowdfunding projects were identified with their corresponding percentages: technology, 20.19%; home appliances, 19.27%; catering, 18.88%; art, 19.97%; and culture, 21.68%.

**Table 1:** Descriptive statistical analysis of key variables.

Variables	Mean	SD	Min	Max	Categorical	Freq.	% of Sample
Crowdfunding performance	1.955	1.515	0.010	11.620	Technology	461	20.190
Video	32.997	68.776	0.000	745	Home appliance	440	19.270
Updates	6.528	7.252	0.000	38	Delicious food	431	18.880
Review	58.489	78.567	0.000	1,332	Art	456	19.970
Crowdfunding experience	19.071	145.982	1.000	840	Culture	495	21.680
Focus	495.475	2037.237	0.000	80,000			
Attention	512.913	1084.993	0.000	3,000			
Goal	72370.910	142493	231	5,000,000			

#### Correlation analysis

Correlation analysis of the eight variables is further conducted. As shown in Table 2, the correlation coefficients between the variables are considerably less than 0.7, indicating that no multicollinearity exists between the variables.

**Table 2:** Correlation matrix analysis of key variable; Pearson’s *r*.

Variables	1	2	3	4	5	6	7	8
1. Crowdfunding performance	1							
2. Video	0.076^***^	1						
3. Updates	0.152^***^	0.017	1					
4. Review	0.246^***^	0.048^**^	0.362^***^	1				
5. Crowdfunding experience	0.085^***^	–0.019	–0.009	0.078^***^	1			
6. Focus	0.086^***^	–0.024	0.161^***^	0.204^***^	–0.002	1		
7. Goal	–0.036^*^	–0.034	0.099^***^	0.184^***^	0.092^***^	0.096^***^	1	
8. Attention	0.122^***^	–0.013	0.225^***^	0.274^***^	0.021	0.504^***^	0.120^***^	1

*** *p* < 0.01,

** *p* < 0.05, and

* *p* < 0.1.

*N* = 2283.

### Research model and empirical results analysis

#### Analysis of empirical results

Hierarchical regression in Stata is performed in this study. The product term is added to analyze the regulating effect of the moderating variable on the independent and dependent variables. Before the product is determined, the average value of the independent and moderating variables is decentralized to minimize the multicollinearity of the variable. Four models are constructed, and the proportion of the actual financing amount to the target amount is considered as the dependent variable for regression analysis of the collected data. The results are listed in Table 3. The VIF values of all variables are less than 10, which verifies that no multicollinearity exists between the variables.

**Table 3:** Analysis of regression results for the effect of high and low signals on financing.

Variables	Model 1		Model 2		Model 3		Model 4
	β,t	VIF	β,t	VIF	β,t	VIF	β,t
Focus	0.026 (1.10)	1.35	0.011 (0.48)	1.42	0.012 (0.56)	1.42	0.012 (0.51)
Goal	–5.594^**^ (–1.90)	1.34	–9.372^**^ (–1.97)	1.35	–1.003^*^ (–1.95)	1.35	–9.923^*^ (–1.91)
Attention	0.015^*^ (1.79)	1.02	0.007 (1.17)	1.24	0.007 (1.16)	1.24	0.069 (1.98)
Video			0.001^**^ (2.34)	1.18	0.001^**^ (2.40)	1.18	0.001^**^ (1.98)
Updates			0.014^***^ (2.76)	1.04	0.014^***^ (2.89)	1.05	0.020^***^ (3.86)
Review			0.004^***^ (7.67)	1.01	0.004^***^ (7.60)	1.01	0.004^***^ (7.53)
Crowdfunding experience					0.008^***^ (3.15)	1.01	0.002^*^ (1.89)
Video*crowdfunding experience							0.003^*^ (1.80)
Updates*crowdfunding experience							0.001^*^ (1.71)
Review*crowdfunding experience							–0.001 (–0.45)
*Cons*	1.903^***^ (40.32)		1.597^***^ (34.74)		1.589^***^ (34.30)		–0.203^***^ (–4.02)
*F*	3.04		21.23		19.91		13.46
Adj-*R*^2^	0.018		0.079		0.085		0.094

*** *p* < 0.01,

** *p* < 0.05, and

* *p* < 0.1.

In Model 1, only control variables are added. Basic information regarding the project (such as the target fundraising amount, the number of likes. and the number of reviews) comprises the control variables, that is, the factors that are confirmed to influence the success of crowdfunding. The results reveal that except for the number of likes, other control variables significantly affect crowdfunding success. This finding is consistent with the existing research results.

Model 2 includes control and independent variables. Independent variables feature low-quality signals such as video duration, the number of updates, and the number of comments on the crowdfunding projects. With the introduction of the independent variables, explanation power of the model is improved, and Adj-*R*^2^ is increased from 0.018 to 0.079. As shown in the table, the video duration (0.001, *p* < = 0.05), the number of updates (0.014, *p* < = 0.05), and the number of comments (0.004, *p* < = 0.01) low-quality signals are positively correlated with crowdfunding success, indicating that H1, H2, and H3 are valid.

**Figure 2:**
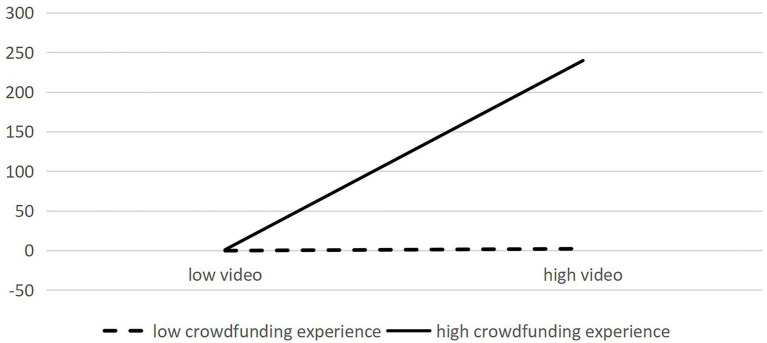
Moderating effect of crowdfunding experience on video duration and crowdfunding success.

**Figure 3:**
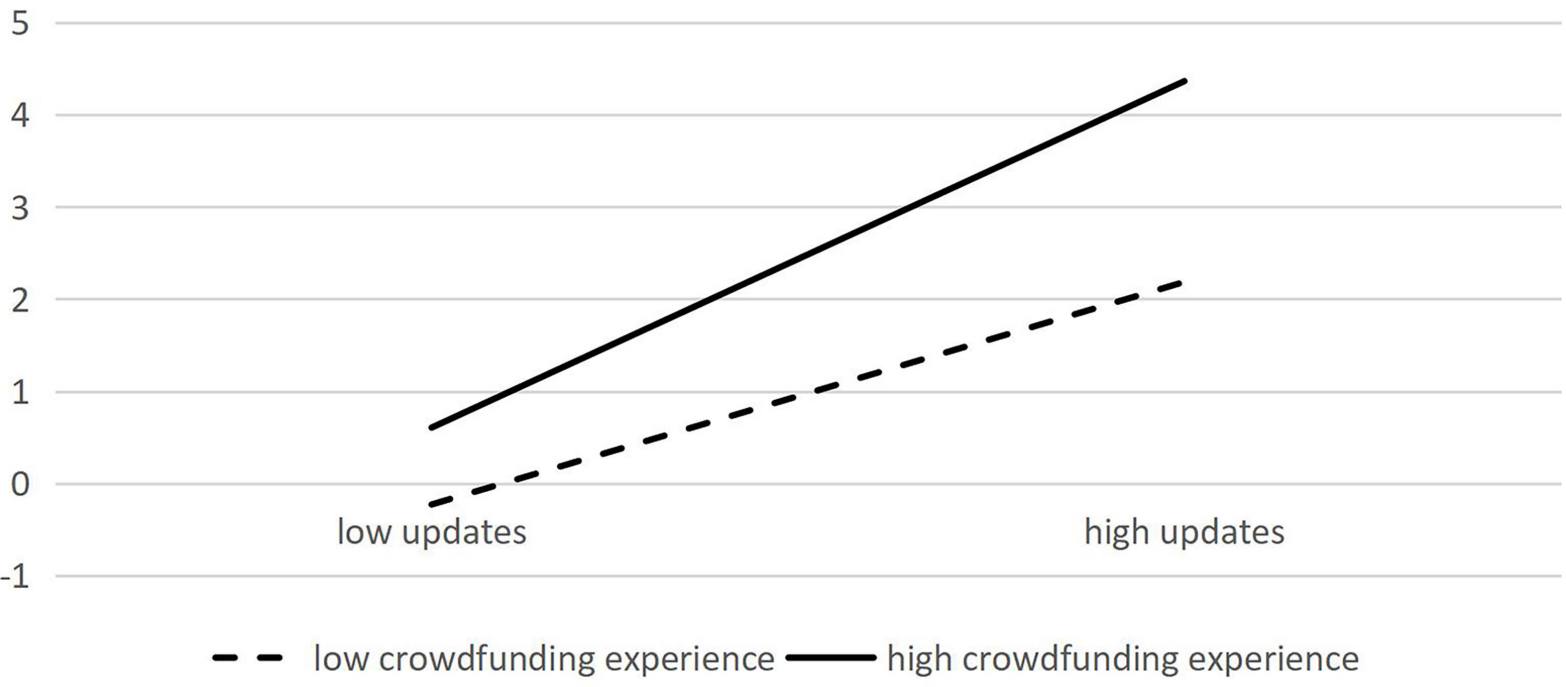
Moderating effect of crowdfunding experience on update quantity and crowdfunding success.

In Models 3-4, apart from the control and independent variables, the crowdfunding experience representing high-quality signals and interactive items of crowdfunding experience with video duration, updates, and comments are incorporated. In Model 4, low-quality signals such as video duration, the number of item updates, and the number of comments, still significantly influence crowdfunding success. Moreover, high-quality signal crowdfunding experience exerts a positive moderating effect on video duration and crowdfunding performance (0.003, *p* < = 0.1) as well as updates and crowdfunding performance (0.001, *p* < = 0.1); however, it has no significant moderating effect on the influence of comments and crowdfunding performance (–0.001, *p* > 0.1), indicating that H4a and H4b are valid, whereas H4c is not valid. Diagrams depicting the moderating effect of crowdfunding experience are presented in Figures 2, 3.

#### Further discussion and analysis

To verify whether the hypotheses in this study are still valid in different types of crowdfunding projects, the overall sample in this study is divided into five sub-samples: technology, home appliances, catering, art, and culture. The functions of the independent, dependent, and moderating variables in different types are listed in Table 4. In technology and home appliances projects, the core variables representing low-quality signals (video duration, number of updates, and number of comments) positively influence crowdfunding success; in the field of catering and culture, only video duration exerts no significant effect on crowdfunding success; in art, the number of updates does not significantly affect crowdfunding success.

**Table 4:** Regression analysis of the effect of differences in project types on crowdfunding.

Variable	Technology	Home appliance	Delicious food	Art	Cultural
Focus	–0.022 (–1.05)	–0.030 (–0.84)	0.033 (0.70)	–0.086^**^ (–2.33)	0.012 (1.24)
Goal	–2.368^**^ (–2.12)	–9.536 (–1.09)	–9.045^**^ (–2.01)	–2.469^*^ (–1.74)	–5.779^***^ (–6.20)
Attention	0.095^***^ (3.93)	0.010^*^ (1.62)	–0.024 (–0.78)	0.011^**^ (3.10)	0.027 (1.35)
Video	0.003^**^ (2.80)	0.280^**^ (2.37)	0.008 (1.03)	–0.004 (–0.29)	0.841 (1.25)
Updates	0.020^**^ (1.85)	0.012^***^ (1.38)	0.040^**^ (2.09)	0.006 (0.29)	0.052^**^ (3.41)
Review	0.004^***^ (4.16)	0.004^**^ (3.78)	0.010^**^ (2.15)	0.003^**^ (2.49)	0.011^***^ (3.82)
Crowdfunding experience	0.004^*^ (1.81)	0.007^**^ (2.13)	0.001 (–0.85)	0.005 (0.60)	0.006^**^ (2.10)
Video*crowdfunding experience	0.020^**^ (2.19)	0.029^**^ (2.14)	0.011^**^ (2.94)	–0.002 (–0.56)	–0.012 (–0.32)
Updates*crowdfunding experience	–0.005 (–0.81)	0.008^*^ (1.57)	0.009^**^ (2.14)	0.003 (0.51)	0.006 (1.01)
Review*crowdfunding experience	–0.001 (–0.28)	–2.689 (–1.34)	–0.005^**^ (–2.61)	–1.528 (–0.17)	0.002^*^ (1.92)
*Cons*	–0.214 (–1.61)	0.004 (0.06)	0.167 (1.28)	–0.106 (–0.81)	0.162 (1.58)
Adj-*R*^2^	0.291	0.151	0.179	0.165	0.227
*N*	461	440	431	456	495

*** *p* < 0.01,

** *p* < 0.05, and

* *p* < 0.1.

The empirical results of this study are generally consistent with the theoretical assumptions. Several exceptions are listed in Table 5, which are discussed in the following section.

**Table 5:** Comparative analysis of empirical results of gross sample and sub-sample.

Variables	Success of crowdfunding					
	Gross sample	Technology	Home appliance	Cate	Art	Cultural
Video	H1 valid	H1 valid	H1 valid	H1 invalid	H1 invalid	H1 invalid
Updates	H2 valid	H2 valid	H2 valid	H2 valid	H2 invalid	H2 valid
Review	H3 valid	H3 valid	H3 valid	H3 valid	H3 valid	H3 valid
Video*crowdfunding experience	H4a valid	H4a valid	H4a valid	H4a valid	H4a invalid	H4a invalid
Updates*crowdfunding experience	H4b valid	H4b invalid	H4b valid	H4b valid	H4b invalid	H4b invalid
Review*crowdfunding experience	H4c invalid	H4c invalid	H4c invalid	H4c invalid	H4c invalid	H4c valid

As shown in Table 5, video duration as a low-quality signal exerts a positive significant effect on financing performance; however, the results for different industries largely vary: video duration positively influences the overall sample, science and technology, and household appliances projects but not in food, art, and culture projects. The reason may be that differences in product type meet the diverse needs of various consumers. Consumers are often less likely to conduct extensive information search and processing on hedonic products and prefer subjective heuristic emotional processing; meanwhile, they tend to screen useful information on life-related utilitarian products (Ren et al., 2021). This observation indicates that for different products, consumers undergo cognitive processing differently with respect to consumption decision-making.

Product attribute difference is the basic difference between crowdfunding projects. Crowdfunding products in the areas of science and technology and household appliances are quality-standardized products. Investor assessment of the quality of such projects often depends on the information sent by fundraisers: the richer the information, the easier for the project to gain recognition and approval from investors. Therefore, the signal function of crowdfunding projects in the areas of science and technology and household appliances exerts a strong influence on the decision making of investors. Meanwhile, food, art, and culture projects are empirical products. Investors can only perceive and assess the quality of the project products through consumption experience or by directly browsing the comments made by consumers before purchasing the project products. The conclusion that the number of comments on crowdfunding projects positively affects financing performance in the total sample and various subsamples (H3 is tenable) is supported to a certain extent. Moreover, the positive effect of the renewal number of crowdfunding projects, representing low-quality signals, on financing performance is not significant in art projects. The possible explanation is that investor assessment of art projects often requires professional knowledge and experience. A large number of ordinary individuals without investment experience often lack the professional ability to identify the signals displayed and fed back by such crowdfunding projects.

Crowdfunding experience, as a high-quality signal, positively moderate the relationship between the video duration of crowdfunding projects and financing performance. It is established in the overall sample and projects in the areas of science and technology, household appliances, and food but not in cultural and artistic projects. Crowdfunding experience also positively regulates the relationship between the number of updated crowdfunding projects and financing performance, which is established in the overall sample, household appliances, and food projects but not in science and technology, art, and culture projects. The possible explanation is that from the perspective of investor psychology, the behavior of individual investors deviates to a certain degree. Specifically, when the level of investment largely varies, the mentality and behavior of individual investors also exhibit apparent individual heterogeneity. Individual investors with low investment have poor risk awareness. Such investors often participate in investment for speculative purposes, whereas mature investors with high investment fully weigh risks and benefits and treat investment activities rationally. This observation suggests that when the per capital investment of crowdfunding projects is high, its participants often rationally evaluate the quality of the project, that is, they pay attention to the richness of crowdfunding experience: the more crowdfunding experience, the richer the crowdfunding experience of the project, and the easier it is to gain the favor of rational participants. When the per capital investment of the project is low, most investors participating in the project may join crowdfunding with the mentality of “joining the fun,” which is highly speculative (Mollick, 2014; Colombo et al., 2015). On the basis of the sample data in the current study, household appliances and science and technology projects recorded had the highest per capita crowdfunding amounts—140,000 and 80,000, respectively—whereas esthetics and culture projects recorded relatively low per capita crowdfunding amounts—60,000 and 40,000, respectively. Therefore, for household appliances and science and technology projects, the crowdfunding experience of high-quality signals tends to correspondingly enhance the relationship between the introduction of low-quality signals, video duration of crowdfunding projects, and financing performance. The per capita crowdfunding amount of food projects is only 20,000 yuan; however, when it comes to food safety, investors pay more attention to their safety, and crowdfunding experience plays a corresponding enhancing role.

Esthetic and cultural projects are associated with lower investment per capita, and investors are speculative. By contrast, they pay less attention to the crowdfunding experience. Therefore, the crowdfunding experience does not significantly regulate the positive relationship between the introduction of low-quality signals, the video duration of crowdfunding projects, the number of updates of crowdfunding projects, and financing performance.

In addition, crowdfunding experience, representing high-quality signals, positively regulates the relationship between the number of comments on crowdfunding projects and financing performance, which is established in cultural projects but not in the overall sample, household appliances, science and technology, and food and art projects. The reason may be that, unlike the traditional financial market, crowdfunding is a new investment and financing model based on the Internet platform, and its initiation and termination completely depend on the crowdfunding intermediary platform. Crowdfunding investors tend to evaluate the benefits and risks of the project from a more perceptual perspective; consequently, the psychological heterogeneity of investors in the crowdfunding market is also significantly higher than that of traditional investors. The investment a priori beliefs, the amount of information collected, and the heterogeneity of individual crowdfunding participants are highly diversified. Their decisions depend on the results of a multiparty information game. Participants in the crowdfunding market often face a huge flow of information. To form judgments and reach decisions quickly, they usually rely on intuition and their own experience cognitive framework (such as “accessibility”, that is, people rely on easily available information rather than all information for judgment). The video introduction of the crowdfunding project and the number of project updates are intuitively and clearly displayed on the project home page, rendering the information highly accessible. Even in the context of the high crowdfunding experience of fundraisers, participants of such projects may pay more attention to the number of video and project updates, compared with the number of video and project updates, as an important standard for their investment decisions.

#### Robustness analysis

The dependent variable in this study is ratio data; thus, Tobit regression analysis is used to verify the robustness of the model. The specific results are listed in Table 6. The conclusions are the same as Table 3, and all assumptions are verified.

**Table 6:** Analysis of regression results of tobit model.

Variable	Model 1	Model 2	Model 3	Model 4
Focus	0.002 (0.146)	0.001 (0.63)	0.001 (0.71)	0.001 (0.67)
Goal	–5.590 (–2.51)	–9.379^***^ (–4.30)	–1.003^***^ (–4.61)	–9.924^***^ (–4.55)
Attention	0.015^***^ (4.60)	0.071^**^ (2.14)	0.070^**^ (2.12)	0.069^**^ (2.10)
Video		0.001^**^ (3.10)	0.001^**^ (3.19)	0.001^**^ (2.90)
Updates		0.014^**^ (3.08)	0.014^**^ (3.22)	0.020^***^ (4.71)
Review		0.004^***^ (9.77)	0.004^***^ (9.51)	0.004^***^ (9.66)
crowdfunding experience			0.008^***^ (3.86)	0.002^***^ (4.18)
Video*crowdfunding experience				0.003^***^ (4.18)
Updates*crowdfunding experience				0.001^***^ (3.58)
Review*crowdfunding experience				–0.001 (–0.94)
*Pseudo R^2^*	0.005	0.022	0.024	0.027
*Log likelihood*	–4165.673	–4092.377	–4084.967	–4073.183
*N*	2,283	2,283	2,283	2,283

*** *p* < 0.01 and

** *p* < 0.05.

### Research conclusions and implications

#### Research findings

To evaluate the effect of information released by fundraisers on crowdfunding success, this study divides the information into two types—low-quality signals and high-quality signals—based on the difference in the cost of information transmission. Moreover, this study establishes a structural model to explore the mechanism underlying the effect of low-quality signals (video duration, number of updates, and number of comments) on financing performance and the moderating effect of high-quality signals (crowdfunding experience). An empirical analysis of 2,283 crowdfunding projects on the JD.com crowdfunding platform is also conducted. The results show that the video duration, number of updates, and number of comments positively contribute to crowdfunding success. Crowdfunding experience positively moderates the relationship between video duration, number of updates, and crowdfunding success.

#### Theoretical contribution

The main theoretical contributions of this paper are as follows: Firstly, this paper enriches the study of the factors influencing financing performance of crowdfunding companies. Although existing literature has explored the influencing factors of crowdfunding performance from the perspective of project characteristics, it has remained at the level of contextualized characteristics and failed to condense the basic characteristics of projects from a theoretical perspective. This paper introduces signal theory into the study of factors influencing crowdfunding performance, and classifies the information posted by fundraisers on crowdfunding platforms into high-quality signals and low-quality signals in terms of the opportunity attributes in crowdfunding. Based on this, the impact of different signals (video duration, number of updates, and number of comments) on crowdfunding performance and boundary conditions are explored. This not only expands the boundaries of the application of signal theory in crowdfunding financing research, but also provides a new entry point and theoretical perspective for the study of financing performance in the field of Internet finance. Secondly, the existing literature has mostly focused on the social capital of individual promoters, but rarely analyzed the factors influencing the crowdfunding performance of crowdfunding companies at the level of the social networks formed during the crowdfunding process. Finally, this paper uses objective data to measure the signal characteristics of crowdfunding projects, improving the scientificity and accuracy of the conclusions and providing an empirical basis for theory-driven research in the era of big data.

#### Practical implications

The findings of this paper have certain implications for financing practices on crowdfunding platforms: Firstly, fundraisers should attach great importance to the quality signal of their crowdfunding projects, and strengthen the management of official information presentation when crowdfunding projects are published to enhance the objectivity and integrity of project descriptions and make them more convincing. Secondly, fundraisers should strategize the disclosure of their information in a reasonable manner and share the latest developments of their projects in real time to enhance the efficiency of investors in identifying high-quality projects, thus improving the engagement of the capital market with the development of crowdfunding projects. Thirdly, fundraisers should implement differentiated management strategies for projects in different conditions, and should innovate based on investors’ value propositions and the latest market trends to ensure that they can continue to meet the personalized and dynamic needs of investors.

#### Research prospects

This study has several limitations: First, it only presents the domestic crowdfunding website JD.com as an example for empirical research. Therefore, the conclusions and findings may not be universal; in subsequent research, more types of crowdfunding platforms should be given attention. Second, this study uses cross-sectional data. In subsequent research, panel data can be built to further explore the influence of signal information provided by fundraisers in different periods and different projects on investment decisions. Third, although this study proves that low-cost signals can enhance crowdfunding performance, whether the companies with these low-cost signals are regarded as high-quality is unclear. As a result of the widespread existence of low-cost signals in crowdfunding, capital may flow thriftlessly to low-quality enterprises, which can be a waste of resources and weaken the value of crowdfunding. Therefore, future research needs to examine the relationship between enterprise quality and low-cost signals. In addition, future research can test whether low-cost signals can predict other quality indicators, such as the future growth of the company or the subsequent capital raising strategy of professional investors and whether the self-owned funds invested by fundraisers and its structure are also essential factors affecting crowdfunding performance.

## Data availability statement

The original contributions presented in the study are included in the article/Supplementary material, further inquiries can be directed to the corresponding author.

## Author contributions

ZW designed the overall research, collected data, and analyzed data. JD wrote part of the manuscript. SY conducted literature review and helped ZW to analyze data. All authors contributed to the article and approved the submitted version.

## Funding

This paper is financially supported by the National Social Science Fund of China (Award number(s): 19BJL070.

## Conflict of interest

The authors declare that the research was conducted in the absence of any commercial or financial relationships that could be construed as a potential conflict of interest.

## Publisher’s note

All claims expressed in this article are solely those of the authors and do not necessarily represent those of their affiliated organizations, or those of the publisher, the editors and the reviewers. Any product that may be evaluated in this article, or claim that may be made by its manufacturer, is not guaranteed or endorsed by the publisher.

## Footnotes

^1^ https://z.jd.com/sceneIndex.html
